# Characterizing the COVID-19 Infodemic on Chinese Social Media: Exploratory Study

**DOI:** 10.2196/26090

**Published:** 2021-02-05

**Authors:** Shuai Zhang, Wenjing Pian, Feicheng Ma, Zhenni Ni, Yunmei Liu

**Affiliations:** 1 School of Information Management Wuhan University Wuhan China; 2 School of Economics and Management Fuzhou University Fuzhou China

**Keywords:** COVID-19, infodemic, infodemiology, epidemic, misinformation, spread characteristics, social media, China, exploratory, dissemination

## Abstract

**Background:**

The COVID-19 infodemic has been disseminating rapidly on social media and posing a significant threat to people’s health and governance systems.

**Objective:**

This study aimed to investigate and analyze posts related to COVID-19 misinformation on major Chinese social media platforms in order to characterize the COVID-19 infodemic.

**Methods:**

We collected posts related to COVID-19 misinformation published on major Chinese social media platforms from January 20 to May 28, 2020, by using PythonToolkit. We used content analysis to identify the quantity and source of prevalent posts and topic modeling to cluster themes related to the COVID-19 infodemic. Furthermore, we explored the quantity, sources, and theme characteristics of the COVID-19 infodemic over time.

**Results:**

The daily number of social media posts related to the COVID-19 infodemic was positively correlated with the daily number of newly confirmed (*r*=0.672, *P*<.01) and newly suspected (*r*=0.497, *P*<.01) COVID-19 cases. The COVID-19 infodemic showed a characteristic of gradual progress, which can be divided into 5 stages: incubation, outbreak, stalemate, control, and recovery. The sources of the COVID-19 infodemic can be divided into 5 types: chat platforms (1100/2745, 40.07%), video-sharing platforms (642/2745, 23.39%), news-sharing platforms (607/2745, 22.11%), health care platforms (239/2745, 8.71%), and Q&A platforms (157/2745, 5.72%), which slightly differed at each stage. The themes related to the COVID-19 infodemic were clustered into 8 categories: “conspiracy theories” (648/2745, 23.61%), “government response” (544/2745, 19.82%), “prevention action” (411/2745, 14.97%), “new cases” (365/2745, 13.30%), “transmission routes” (244/2745, 8.89%), “origin and nomenclature” (228/2745, 8.30%), “vaccines and medicines” (154/2745, 5.61%), and “symptoms and detection” (151/2745, 5.50%), which were prominently diverse at different stages. Additionally, the COVID-19 infodemic showed the characteristic of repeated fluctuations.

**Conclusions:**

Our study found that the COVID-19 infodemic on Chinese social media was characterized by gradual progress, videoization, and repeated fluctuations. Furthermore, our findings suggest that the COVID-19 infodemic is paralleled to the propagation of the COVID-19 epidemic. We have tracked the COVID-19 infodemic across Chinese social media, providing critical new insights into the characteristics of the infodemic and pointing out opportunities for preventing and controlling the COVID-19 infodemic.

## Introduction

### Background

As the COVID-19 pandemic continued to develop, we experienced the parallel rise of the COVID-19 infodemic [1,2]. This infodemic is a phenomenon of overabundance of information caused by COVID-19 misinformation, which has rapidly propagated on social media and attracted widespread attention from the government and health agencies during the ongoing pandemic [3,4]. The infodemic has made the pandemic worse, harmed more people, and jeopardized the global health system’s reach and sustainability [5,6]. Thus, the World Health Organization (WHO) has called it a disease accompanying the COVID-19 epidemic [7].

The term “infodemic” is derived from a combination of the root words “information” and “epidemic” and was coined by Eysenbach in 2002 [8], when a SARS outbreak had emerged in the world. It was not until the WHO Director-General reintroduced the term “infodemic” at the Munich Security Conference on February 15, 2020, that it had begun to be used more widely, summarizing the challenges posed by COVID-19 misinformation to our society [9]. In this study, the term “infodemic” refers to an information abundance phenomenon wherein the lack of reliable, trustworthy, and accurate information associated with the COVID-19 epidemic has enabled COVID-19 misinformation to disseminate rapidly across a variety of social media platforms [10]. Thus, the COVID-19 infodemic is also called the COVID-19 misinformation epidemic [11].

Misinformation refers to a claim that is not supported by scientific evidence and expert opinion [12]. This definition explains that misinformation can act as an umbrella concept to explain different types of incorrect information, such as false information, fake news, misleading information, rumors, and anecdotal information, regardless of the degree of facticity and deception [13]. Research linking misinformation to epidemic diseases is emerging [14]. There have been multiple instances where misinformation has been correlated with negative public health outcomes, including the spread of Zika virus [15] and vaccine-preventable infectious diseases in many countries worldwide [16]. Another salient example is the COVID-19 pandemic. For instance, Nsoesie and Oladeji [17] investigated the impact of misinformation on public health during the COVID-19 pandemic. They found that COVID-19 misinformation prevented people from demonstrating effective health behaviors and weakened the public’s trust in the health care system. Therefore, dealing with COVID-19 misinformation requires urgent attention.

The increasing global access of social media via mobile phones has led to an exponential increase in the generation of misinformation as well as the number of possible ways to obtain it, thus resulting in an infodemic. Infodemics have co-occurred with epidemics such as Ebola and Zika virus in the past [18,19]. However, the COVID-19 infodemic is significantly different from the earlier ones. It has been reported as “the first true social-media infodemic” [20]. It is also the first infodemic to have been disseminated widely through social media and has significantly impacted public health [21]. By the beginning of 2020, more than 3.8 billion people used social media [22]. Moreover, social media is one of the most popular media for information dissemination and distribution, with 20%-87% usage surging during the crisis [23]. Recently, Oxford’s Reuters Institute investigated the dissemination of misinformation and found that a majority (88%) of the misinformation about COVID-19 originated from social media [24]. In Italy, approximately 46,000 posts posted per day on social media in March 2020 were linked to COVID-19 misinformation [25].

In China, the COVID-19 infodemic was more serious [26]. Two-thirds of the Chinese population used social media, and approximately 87% of all users encountered relevant misinformation during the COVID-19 crisis [27]. Examples of such misinformation spread on Chinese social media include that compound Chinese medicine and Banlangen could cure COVID-19; consuming methanol, ethanol, or bleach could protect or cure COVID-19; pneumonia vaccines could protect against SARS-CoV-2; eating garlic could kill the virus; and 5G mobile network has spread COVID-19 [28]. Moreover, China was the first country to experience the COVID-19 infodemic [18]. In December 2019, the first case of COVID-19 was reported in China [29]. In subsequent weeks, the rapid spread of novel coronavirus caused increasing discussion among social media users. Countless unproven stories, advice, and therapies related to COVID-19 were prevalent and skyrocketed on Chinese social media platforms [30].

The COVID-19 infodemic is immensely concerning because all social media users can be affected by it, which poses a severe threat to public health [31]. A study showed that 5800 people were admitted to the hospital as a result of the COVID-19 misinformation disseminated on social media [32]. More seriously, the misinformation that consumption of neat alcohol can cure COVID-19 led to hundreds of deaths due to poisoning [33]. Moreover, the infodemic on social media can also lead to inappropriate actions by users and endanger the government and health agencies' efforts to manage COVID-19, inducing panic and xenophobia [2,34].

Given the negative impact of the COVID-19 infodemic on social media, especially on Chinese social media, the government and health agencies need to assess the COVID-19 infodemic on Chinese social media. Therefore, in this study, we aimed to analyze the quantity, source, and theme characteristics of the COVID-19 infodemic by collecting posts related to COVID-19 misinformation on published on Chinese social media from January 20 to May 28, 2020. Specifically, we used content analysis to analyze the quantity and source of the COVID-19 infodemic. Then, we used topic modeling to analyze various themes of the infodemic. Finally, we explored the quantity, source, and theme characteristics of the COVID-19 infodemic over time.

### Prior Works

Previous studies have investigated the distribution and themes of infodemics on social media in other countries. For example, Oyeyemi et al [35] used the Twitter search engine to collect posts about the Ebola virus from September 1 to 7, 2014. They found that 58.9% of the posts were identified as misinformation. Moreover, the study indicated that misinformation was rampant on social media and had a greater impact on users than did correct information. Similarly, Tran and Lee [36] investigated the propagation of the Ebola infodemic and found that misinformation was more widespread on social media than correct information. Glowacki et al [37] further collected posts about the Zika virus on the live Twitter chat initiated by the Centers for Disease Control and Prevention. They applied topic modeling and derived the following 10 topics relevant to the Zika epidemic: “virology of Zika,” “spread,” “consequences for infants,” “promotion of the chat,” “prevention and travel precautions,” “education and testing for the virus,” “consequences for pregnant women trying to conceive,” “insect repellant,” “sexual transmission,” and “symptoms.”

With the world’s commitment to the fight against COVID-19, there has been active research in many areas, including social media and quantitative analyses. For example, Kouzy et al [11] assessed the source characteristics of the COVID-19 infodemic being spread on Twitter. They used descriptive statistics to analyze Twitter accounts and post characteristics and found that 66% of misinformation posts regarding the COVID-19 epidemic was posted by unverified individual or group accounts, and 19.2% were posted by verified Twitter users’ accounts. Moreover, they indicated that the COVID-19 infodemic is being propagated at an alarming rate on social media. Another study by the COVID-19 Infodemic Observatory found that robots generated approximately 42% of the social media posts related to the pandemic, of which 40% were considered unreliable [38]. Similarly, the Bruno Kessler Foundation analyzed 112 million social media posts about COVID-19 information [26]. The results showed that 40% of this information was from unreliable sources [22]. At the same time, Moon et al [39] collected 200 of the most viewed Korean-language YouTube videos on COVID-19 published from January 1 to April 30, 2020. They found that 37.14% of the videos contained misinformation, and independent videos generated by the user showed the highest proportion of misinformation at 68.09%, whereas all government-generated videos were regarded as useful. Additionally, Naeem et al [23] selected 1225 pieces of misinformation about COVID-19 published in the English language on various social media platforms from January 1 to April 30, 2020, and coded the data using an open coding scheme. They concluded that the theme characteristics of the COVID-19 infodemic include “false claims,” “half-backed conspiracy theories,” “pseudoscientific therapies,” “regarding the diagnosis,” “treatment,” “prevention,” “origin,” and “spread of the virus.”

### Objectives

An increasing number of studies have begun to highlight the COVID-19 infodemic on social media. However, attempts to characterize the spread of the COVID-19 infodemic on social media, especially on Chinese social media platforms, are currently lacking. Hence, in this study, we used content analysis and topic modeling to analyze the COVID-19 infodemic across Chinese social media platforms to gain new insights into the quantity, source, and theme characteristics of the infodemic over time and propose measures to contain the dissemination of misinformation during the COVID-19 infodemic.

## Methods

### Data Collection

The database for this study was obtained from Qingbo Big Data Agency [40], which covers data from almost all major Chinese social media platforms, such as WeChat, Weibo, and TikTok. The posts collected included microblogs, messages, or short articles shared on these social media platforms. Our search strategy to retrieve post data comprised of the following keywords in Chinese: “coronavirus,” “2019-nCoV,” “COVID-19,” “corona,” “new pneumonia,” and “new crown.” We used PythonToolkit to crawl the data searched using the abovementioned keywords from January 20 to May 28, 2020. The data collection process was as follows. First, we searched the Qingbo Big Data Agency to obtain the results page. Second, the web link crawler was initiated, and the title and URL fields of all web pages were collected. Third, these fields were stored in the url_list dataset of the MongoDB database. Fourth, the web page details crawler was launched, the post published time, source, and text fields of the details page were collected. Finally, these fields were stored in the info_list dataset of the MongoDB database. After data collection was completed, datasets url_list and info_list from the MongoDB database were exported. It should be noted that for video-sharing platforms, the textual description of the video was captured as the post data. Data collection began on January 20, 2020, when the Chinese State Council officially announced the COVID-19 epidemic as a public health emergency [31]. Data collection ended on May 28, 2020, when the National Health Commission of the People’s Republic of China issued that the number of new confirmed cases and new suspected cases of COVID-19 in China was zero for the first time. This data collection period could reflect the overall spread of the COVID-19 infodemic on Chinese social media.

All data regarding COVID-19 posts were retrieved, and 723,216 posts were extracted in total. To improve the representativeness of data, we removed incomplete data from the fields and deleted text longer than 400 Chinese characters [41], thus obtaining data from a total of 143,197 posts. Because most of these posts were reposts, we only retained 19,188 of the original post data. We verified the authenticity of post data using the following 2 steps. First, we conduct fact-checking according to the authority organization, such as the National Health Commission of the People’s Republic of China, the Chinese Center for Disease Control and Prevention, and the Cyberspace Administration of China. We only retained those posts that were judged to be fake and obtained data from 1729 posts. Next, two independent researchers reviewed and evaluated the remaining posts. One of them is a doctoral student in Library and Information Science, and the other has a bachelor’s degree in Medicine. Discrepancies between the 2 researchers were resolved through mutual discussion. The Cohen kappa coefficient was used to analyze the interreviewer reliability for coding. Cohen’s Kappa value for the 2 researchers was 0.79, suggesting substantial agreement between them [42]. Ultimately, we obtained 2745 posts related to COVID-19 misinformation as the final analysis sample for this study, which was the largest dataset the study team could obtain with the available resources. The post data was organized and stored chronologically, and the title, URL, post date, source, and text were recorded. Table 1 details the data format of the posts collected for the analysis.

**Table 1:** Data format of COVID-19 misinformation posts (partial) on Chinese social media.

Title	URL	Post date	Source	Text
Reposting well-known! One article to understand the new coronavirus…	https://mp.weixin.qq.com/s?src=11&timestamp=1598007513&ver…	2020-01-20	WeChat	…Wuhan virus is the long-standing SARS Coronavirus…
Highly concerned! Wuhan pneumonia continues to spread, 2 cases in Beijing and 1 case in Shenzhen, the public should…	https://mp.weixin.qq.com/s?src=11&timestamp=1598005483&ver…	2020-01-20	WeChat	…WeChat users, who claim to be medical staff, said: “there are several cases in our hospital, which have been strictly isolated. 80% of the cases are said to be SARS case”…
Reposted from Weibo by Cui Tiange, a North American bioinformatics researcher: the new crown virus…	https://m.weibo.cn/status/4463141235003931?sudaref…	2020-01-21	Weibo	…The “mysterious disease” in Wuhan has been confirmed as a new type of SARS virus, or the similarity between Wuhan virus and SARS is as high as 90%…
Weibo_#Academician Zhong Nanshan's team recommends saltwater gargle antivirus#…	https://weibo.com/5044281310/IqH405BUW?type=comment….	2020-01-22	Weibo	…Academician Zhong Nanshan suggests that saltwater gargle prevent new coronavirus…
Six latest facts about Wuhan pneumonia…	https://zhuanlan.zhihu.com/p/103781132…	2020-01-22	Zhihu	…Wuhan virus is a new type of SARS virus. SARS has not disappeared and has been parasitic in bats…
Burst! A patient with “Wuhan pneumonia” fled from Peking Union Medical College Hospital…	http://news.sina.com.cn/c/2020-01-22/doc-iihnzhha4099491…	2020-01-22	Sina	…A patient identified as “Wuhan pneumonia” escaped from Peking Union Medical College Hospital and lost contact…

### Data Processing

We used Python (version 3.8.5) and SPSS software (version 25.0; IBM Corp) to perform all data processing and analyses. Time segmentation adapted from the practice of Zhao et al [18] was used to divide the period into 19 time segments (T_1_: January 20-26, 2020; T_2_: January 27 to February 2, 2020; T_3_: February 3-9, 2020; T_4_: February 10-16, 2020; T_5_: February 17-23, 2020; T_6_: February 24 to Mar 1, 2020; T_7_: March 2-8, 2020; T_8_: March 9-15, 2020; T_9_: March 16-22, 2020; T_10_: March 23-29, 2020; T_11_: March 30 to April 5, 2020; T_12_: April 6-12, 2020; T_13_: April 13-19, 2020; T_14_: April 20-26, 2020; T_15_: April 27 to May 3, 2020; T_16_: May 4-10, 2020; T_17_: May 11-17, 2020; T_18_: May 18-24, 2020; and T_19_: May 25-28, 2020). Among these segments, the last time segment is 4 days long, and the other time segments are 7 days long each, with the total period spanning 130 days.

Based on the classification of social media websites by the CNNIC (China Internet Network Information Center) [43], the sources of posts were categorized into 5 types: chat platforms, video-sharing platforms, news-sharing platforms, health care platforms, and Q&A platforms. The chat platforms included WeChat, Weibo, and QQ. The video-sharing platforms included TikTok, Kuaishou, and Pear Video. The news-sharing platforms included Toutiao, Sina, and Tencent. The health care platforms included DXY.cn, Haodf.com, and Chunyu Yisheng. The Q&A platforms include Zhihu, Douban, and Jianshu (see a full list of Chinese social media types and major social media sites in Multimedia Appendix 1).

The “jieba” package in Python was used to segment post text. We limited the parts of speech of the post text to 9 categories (“n,” “nr,” “ns,” “nt,” “eng,” “v,” “vn,” “vs,” and “d”). We adapted the method described by Medford et al [29] to merge synonyms into a unified form (eg, “disinfectant powder” and “disinfectant water” into “disinfectants” and “suspense of business” and “termination of business” into “close down”). The Gensim package in Python was used to perform latent Dirichlet allocation (LDA) model. A post contains only one dominant topic. We used different numbers of topics to iteratively train multiple LDA models to maximize the topic coherence score. After more than 10 tests, the results with the highest coherence score in the use of the LDA model with 8 topics were selected. Each topic contains 15 words adhering to convention and is manually tagged with a theme.

### Data Analysis

We explored characteristics of the COVID-19 infodemic on Chinese social media from the perspective of quantity, source, and theme. From the perspective of quantity, we counted the daily number of posts and obtained the number of newly confirmed cases and suspected cases each day from the official website of the Chinese Center for Disease Control and Prevention. We performed Pearson correlation analysis to explore the relationship between the daily number of posts with the number of newly confirmed cases and suspected cases per day. Moreover, we calculated the maximum, minimum, upper quartile, lower quartile, and median number of posts in each time segment, and we visualized them to intuitively evaluate the characteristics of post propagation. From the perspective of source and theme, we calculated the sources and themes of posts based on the number of occurrences. Additionally, we visualized the number of sources of posts in each time segment to analyze the source characteristics of the COVID-19 infodemic. We then created a visualization of the time segment of themes of posts to assess the change in themes over time.

## Results

### Quantity Characteristics

**Figure 1:**
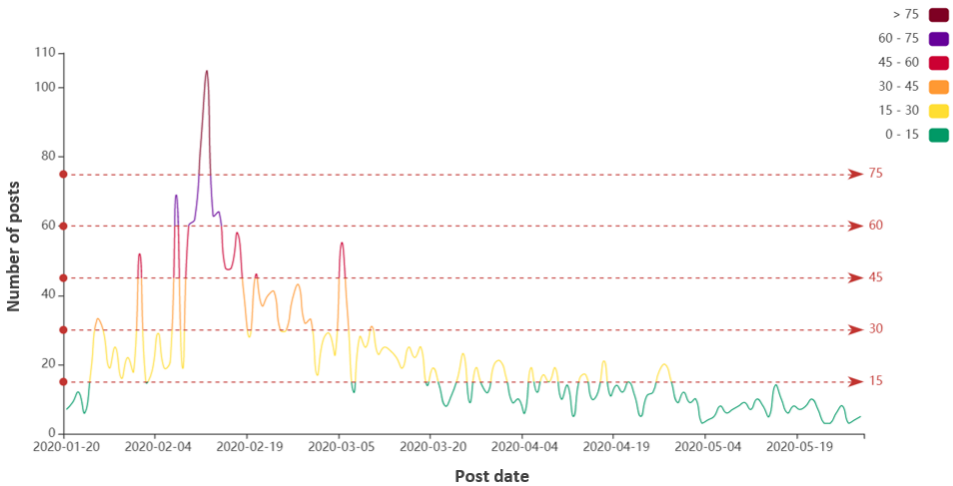
Daily number of posts related to COVID-19 misinformation on Chinese social media platforms. Different colored lines indicate the number of posts published.

Figure 1 shows the daily number of posts related to the COVID-19 misinformation on Chinese social media that was published from January 20 to May 28, 2020. The maximum number of posts published in a day was 105, whereas the minimum number was 3 (mean 21.12, SD 17.35). Pearson correlation analysis shows that the daily number of posts related to the COVID-19 infodemic was positively correlated with the daily number of newly confirmed (*r*=0.672, *P*<.01) and newly suspected (*r*=0.497, *P*<.01) COVID-19 cases in China. In other words, the more posts related to the COVID-19 misinformation that were published per day, the greater was the severity of the COVID-19 epidemic, and vice versa.

**Figure 2:**
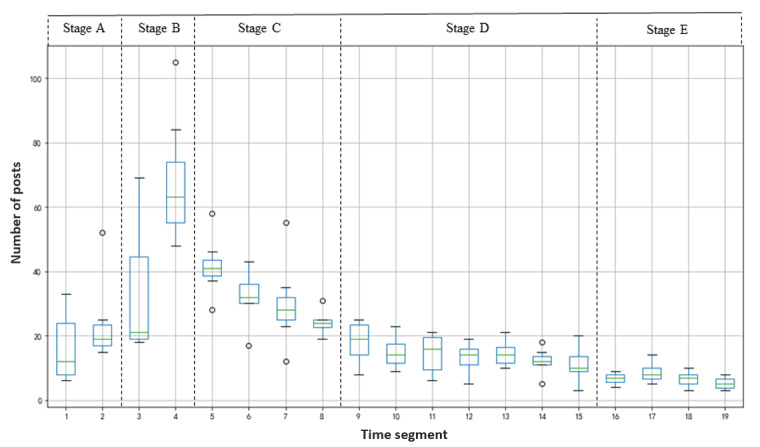
Box plot of the number of social media posts in each time segment.

We used a box plot to describe the spread of social media posts according to different time segments (Figure 2). We found that the posts presented a spread characteristic indicating gradual progress. That is, the number of posts first increases slowly with the time segment, then concentrates on the burst, and then moderates gradually as the time segments continue to advance. Furthermore, the COVID-19 infodemic on Chinese social media can be divided into 5 periods (see Table 2). During the incubation period (Stage A: T_1_-T_2_), the number of posts showed slow growth, with the mean and median values of approximately 20 per day. Then, the number of posts rapidly increased during the outbreak period (Stage B: T3-T4), and the mean and median values soared to approximately 50 per day. During the stalemate period (Stage C: T_5_-T_8_), the number of posts remained at a high level, and the mean and median values were approximately 30 per day. During the control period (Stage D: T_9_-T_15_), the number of posts dropped significantly, with mean and median values of approximately 14 per day. Finally, the number of posts has decreased sluggishly in the recovery period (Stage E: T_16_-T_19_), and the mean and median values remained at approximately 7 per day.

**Table 2:** Periods of the COVID-19 infodemic based on data from relevant Chinese social media posts.

Post metric	Incubation period	Outbreak period	Stalemate period	Control period	Recovery period
Time segment	T_1_-T_2_	T_3_-T_4_	T_5_-T_8_	T_9_-T_15_	T_16_-T_19_
Mean (SD) (days)	20.14 (11.72)	50.64 (25.89)	31.89 (10.56)	14.02 (5.18)	6.69 (2.55)
Range	6-52	18-105	12-58	3-25	3-14
Median (IQR) (days)	18 (12.75-24.25)	54 (23-63.75)	30 (24.75-39.25)	14 (10-18)	7 (5-8)

### Source Characteristics

**Figure 3:**
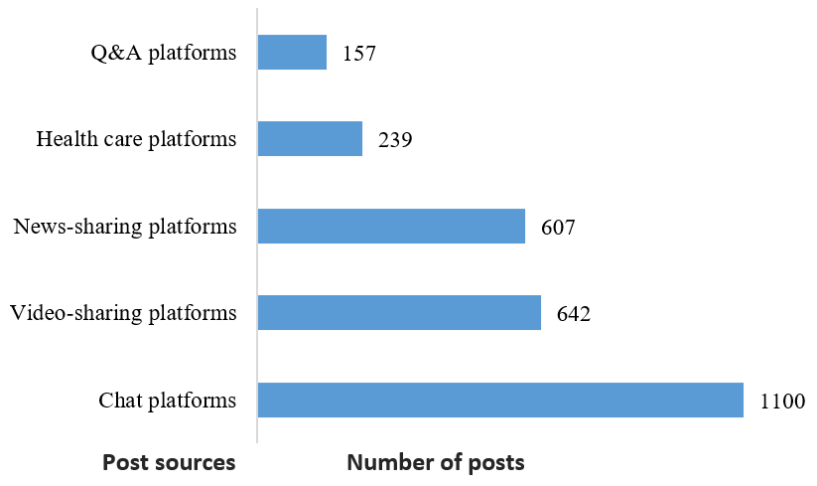
Sources of posts about COVID‐19 misinformation on various Chinese social media platforms.

Of the posts related to the COVID-19 misinformation that were classified (Figure 3), chat platforms (1100/ 2745, 40.07%) represented the largest source of the COVID‐19 infodemic, followed by video-sharing platforms (642/ 2745, 23.39%) and news-sharing platforms (607/2745, 22.11%). The proportions of health care platforms (239/2745, 8.71%) and Q&A platforms (157/2745, 5.72%) were relatively small.

**Figure 4:**
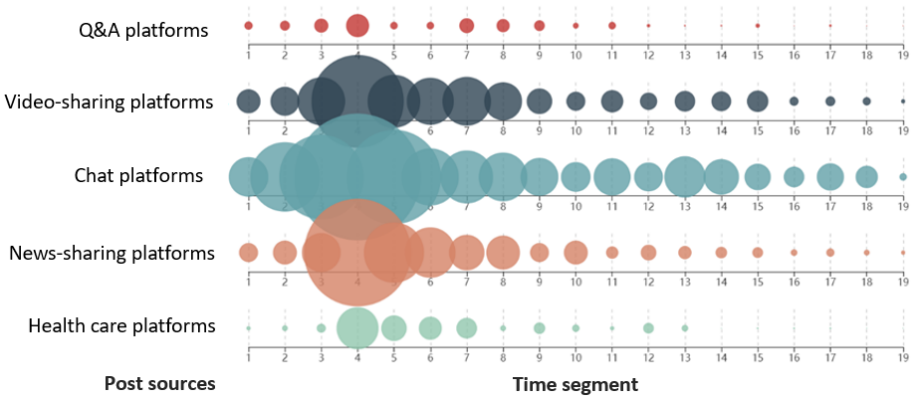
Number of sources of social media posts in each time segment. Different colored dots represent different sources, and their sizes represent the proportion of sources.

We visualized the number of sources of posts in each time segment (Figure 4). Chat, video-sharing, and news-sharing platforms were the main sources for the spread of posts during the incubation period (T_1_-T_2_). Then, the posts began to spread toward the health care and Q&A platforms during the outbreak period (T_3_-T_4_). Thereafter, the posts were broadly spread on all social media platforms and were maintained at a high level during the stalemate period (T_5_-T_8_). During the control period (T_9_-T_15_), the spread of the posts on chat and video-sharing platforms alternately increased and decreased, whereas the spread of posts on news-sharing, health care, and Q&A platforms evidently declined. Finally, the spread of posts on chat platforms also gradually decreased during the recovery period (T_16_-T_19_), and the spread on other social media platforms dropped sharply and remained at a low level.

### Theme Characteristics

**Figure 5:**
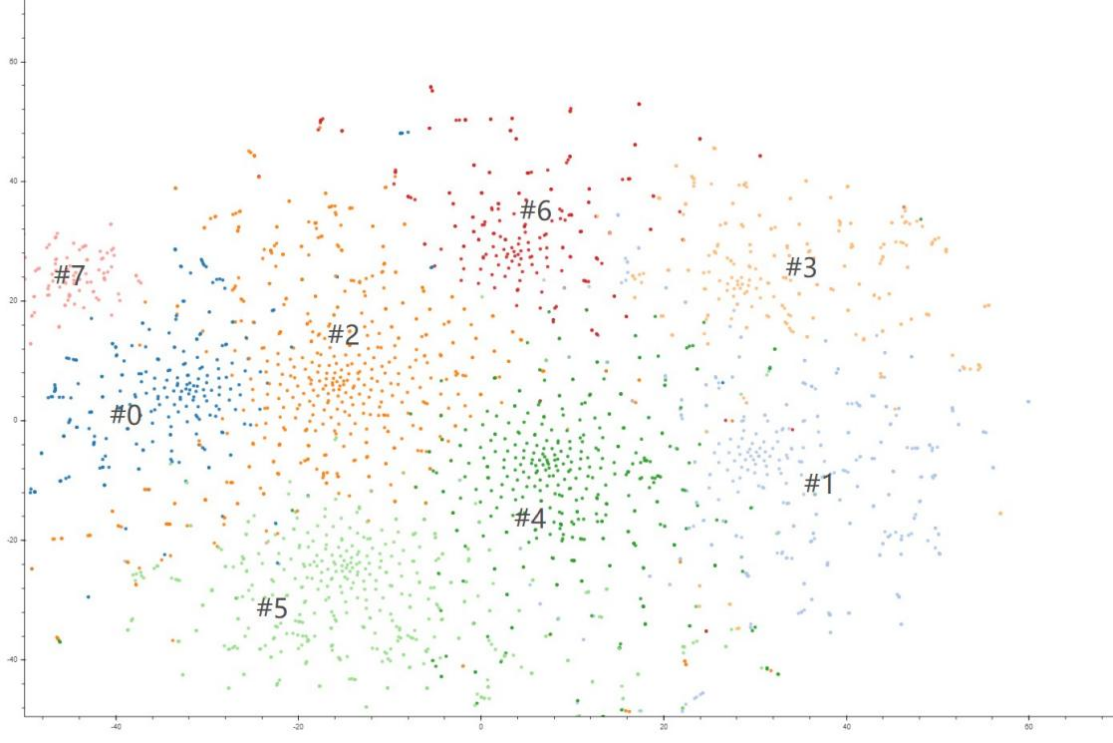
Visualization of themes identified by latent Dirichlet allocation.

Topic modeling identified 8 different themes, which are illustrated in Figure 5. The 15 keywords that contributed to each theme with their potential theme labels are shown in Table 3. Based on LDA analysis, we obtained a specific theme for each post. The popularity of each theme was determined based on the proportion of posts in each theme considering the overall post data. The most common primary theme was “conspiracy theories” (648/2745, 23.61%), which included topics such as “Academician Zhong Nanshan did not wear a mask for rounds,” “Academician Li Lanjuan helped her son sell medicines,” “Dr. Li Wenliang danced before his death,” and “Wuhan Huoshenshan was designed by the Japanese.” The second most common theme was “government response” (544/2745, 19.82%), which included the following topics: “The city would be closed down at 2:00 PM on January 25, 2020, in Xinyang, Henan province;” “Wuhan gas stations would be closed;” and “Jingzhou, Hubei Province, would suspend issuing permits for leaving Hubei Province.” Thereafter, the themes discussed were “prevention action” (411/2745, 14.97%) and “new cases” (365/2745, 13.30%), which included topics such as “Wearing multi-layer masks can prevent the virus,” “Smoking vinegar can prevent the virus,” “Six promoters of Wuhan Zhongbai Supermarket were confirmed with novel coronavirus pneumonia,” and “More than 20,000 new confirmed close contacts in Qingdao.” The other common themes included “transmission route” (244/2745, 8.89%) as well as “origin and nomenclature” (228/2745, 8.30%). These themes included the following topics: “Catkins can transmit COVID-19,” “COVID-19 is a biological weapon,” and “COVID-19 was made by the laboratory.” Other themes included “vaccines and medicines” (154/2745, 5.61%) as well as “symptoms and detection” (151/2745, 5.50%), which included topics such as “CT image is used as the latest standard for judging the diagnosis of COVID-19,” “Hold your breath for 10 seconds to test whether you are infected with the virus,” “The first COVID-19 vaccine was successfully developed and injected,” and “Hydroxychloroquine and chloroquine are specific drugs for COVID-19.”

**Table 3:** Theme labels and keywords contributing to the topic model.

Theme labels	Keywords contributing to topic model
Origin and nomenclature (#0)	COVID-19, SARS, Corona, SARI, host animals, bat, pangolin, variation, pestilence, influenza, the natural world, man-made, biological weapon, laboratory, patient zero
Transmission routes (#3)	5G, seafood, aerosol, catkin, mosquito, paper money, tap water, aquatic product, public toilet, sweater, air conditioner, pet dog, freshwater fish, salmon, subway ticket
Prevention action (#4)	prevention, face mask, disinfectant, alcohol, N95, chlorine, liquor, onion, garlic, vinegar, tea, smoke, strawberries, eyedrops, balm
New cases (#1)	infection, case, confirmed, suspected, patient, isolation, hospital, community, airport, hotel, school, nursing home, student, old people, infant
Symptoms and detection (#7)	detection, test positive, cough, fever, outpatient, computed tomography, lung, blood type, plasma, antibody, diagnostic kit, self-test, suffocation, asymptomatic, expectoration
Government response (#5)	lockdown, road closure, close down, health code, living material, trip, network, transportation, traffic control, home quarantine, traffic permitting, work resumption, school opens, customs office, inbound
Vaccines and medicines (#6)	vaccine, chloroquine, remdesivir, azithromycin, Shuanghuanglian oral liquid, Lianhua Qingwen capsule, Banlangen, oseltamivir, azithromycin, aspirin, Angong Niuhuang Wan, traditional Chinese medicine, Bacillus Calmette-Guerin, toxic strain, Chinese fevervine herb
Conspiracy theories (#2)	Zhong Nanshan, Li Lanjuan, Li Wenliang, Leishenshan, Huoshenshan, Donald John Trump, modular hospital, doctors, nurses, online course, blood donation, suicide, escape, medical corps, cleaner, Red Cross Society

**Figure 6:**
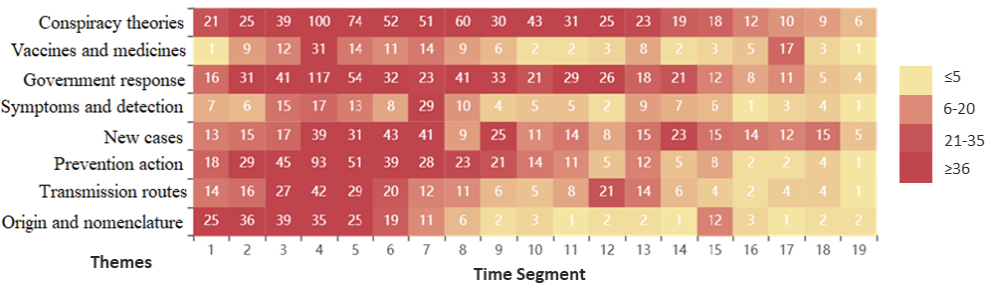
Heat map of themes related to the COVID-19 infodemic according to time segments. Data within the figure represent the number of posts per theme in each time segment. Individual values in the matrix are represented in different background colors according to the number of posts (range) on a particular theme in that time segment.

The “Pyecharts” package in Python was used to draw a heat map of themes according to the time segments (Figure 6). We found that different hot themes were discussed at each stage of the COVID-19 infodemic. The theme “origin and nomenclature” was discussed from the start of the incubation period (T_1_-T_2_). The themes “government response,” “new cases,” and “transmission routes” were debated on social media during the outbreak period (T_3_-T_4_). The discussion of “conspiracy theories” and “symptoms and detection” increased significantly in the stalemate period (T_5_-T_8_). During the control period (T_9_-T_15_), the discussion of “prevention action” was concentrated. Subsequently, the theme “vaccines and medicines” was the focus of discussion on social media during the recovery period (T_16_-T_19_).

We further found that the COVID-19 infodemic presented a spread characteristic of repeated fluctuations across time segments. As shown in Figure 6, each theme is repeated in the time segment, and the theme discussion rate gradually decreases. For example, the theme “government response” not only appeared in the time segment T_2_-T_6_, but it was also spread in the time segment T_8_-T_9_, T_11_-T_12_, T_14_, and T_17_. Moreover, we determined the number of repeated posts for each theme in the time segment (see Table 4) and calculated that the total ratio of repeated posts to be 0.2849 (782/2745), which means that 28.49% of the posts were posted repeatedly in various time segment. This once again verified the spread characteristic of the COVID-19 infodemic that fluctuates repeatedly across time segments. Additionally, the repetition percentage of the themes “conspiracy theories” (198/648, 30.6%), “new cases” (110/365, 30.1%), and “prevention action” (121/411, 29.4%) were particularly high, followed by the themes “government response” (157/544, 28.9%), “origin and nomenclature” (63/228, 27.6%), and “transmission routes” (64/244, 26.2%). The repetition percentage of the themes “vaccines and medicines” (37/154, 24%) and “symptoms and detection” (32/151, 21.2%), however, were relatively low. Differences in repetition among the themes were analyzed by analysis of variance and post hoc analysis, which revealed significant differences in the repetition of themes (F=2.402, *P*=.02). The post hoc tests showed that the theme of “conspiracy theories” was more significant than the theme “symptoms and detection” (*P*<.01) and the theme “vaccines and medicines” (*P*=.04). However, no significant differences were observed between the themes “symptoms and detection” and “vaccines and medicines” (*P*=.29).

**Table 4:** Percentage of repeated posts categorized by themes.

Theme categories	Number of posts	Number of repeated posts	Repeated posts (%)
Conspiracy theories	648	198	30.56
Vaccines and medicines	154	37	24.03
Government response	544	157	28.86
Symptoms and detection	151	32	21.19
New cases	365	110	30.14
Prevention action	411	121	29.44
Transmission routes	244	64	26.23
Origin and nomenclature	228	63	27.63
Total	2745	782	28.49

## Discussion

### Principal Findings

To our knowledge, this study is the first of its kind to analyze posts related to the COVID-19 infodemic on Chinese social media platforms. Previous studies about the COVID-19 infodemic on social media have been mainly qualitative in nature [1,7]. In this study, we analyzed 2745 posts about the COVID-19 infodemic published on Chinese social media platforms between January 20, 2020, and May 28, 2020, which had more than 100 million views cumulatively. We analyzed various characteristics of the COVID-19 infodemic on Chinese social media from the perspective of quantity, source, and theme, to provide decision support for government and health agencies. Below, we discuss 5 key findings of our study that are noteworthy.

First, it was interesting to find that the daily number of posts related to the COVID-19 misinformation on Chinese social media was positively correlated with the daily number of newly confirmed (*r*=0.672, *P*<.01) and newly suspected (*r*=0.497, *P*<.01) COVID-19 cases in China. This finding indicated that the COVID-19 infodemic paralleled the propagation of the COVID-19 outbreak in China. Our finding is similar to previous studies on posts related to the H7N9 outbreak on Weibo, which showed a positive correlation between the daily number of posts published and the daily number of deaths due to H7N9 infection [44].

Second, we found that the COVID-19 infodemic was characterized by gradual progress, which can be divided into 5 stages. During the incubation period (T_1_-T_2_), since COVID-19 cases were only reported in Wuhan, the COVID-19 infodemic showed slow growth. Subsequently, the COVID-19 infodemic increased rapidly during the outbreak period (T_3_-T_4_), as the COVID-19 began to spread across China, causing a mass of public discussion on social media. Thereafter, as the number of COVID-19 cases continued to increase, the COVID-19 infodemic maintained a high level in the stalemate period (T_5_-T_8_). During the control period (T_9_-T_15_), because of the remarkable decrease in the number of COVID-19 cases, the COVID-19 infodemic also significantly declined. Finally, during the recovery period (T_16_-T_19_), the COVID-19 infodemic generally decreased, as the number of COVID-19 cases dropped constantly.

Third, our study found that the COVID-19 infodemic was characterized by videoization. Sources of the COVID-19 infodemic can be divided into 5 types (ie, chat, video-sharing, news-sharing, health care, and Q&A platforms). Among these, video-sharing platforms (23.38%) emerged as the second-largest source after chat platforms. The dissemination mode of “seeing is believing” was subduing public awareness of the COVID-19 epidemic. Moreover, it may be a new spread characteristic for the infodemic. Additionally, we found that the COVID-19 infodemic was more prevalent on chat, video-sharing, and news-sharing platforms than on health care and Q&A platforms. One possible explanation for this difference is that on chat, video-sharing, and news-sharing platforms, users tend to post personal experiences more centrally, which may often be inaccurate, whereas more professional expertise may likely be shared on health care and Q&A platforms.

Fourth, we found that the themes of the COVID-19 infodemic changed with different spread characteristics across stages. Users posted a large number of posts about “origin and nomenclature” in the incubation period (T_1_-T_2_) and gradually changed to themes such as “government response,” “new cases,” and “transmission routes” in the outbreak period (T_3_-T_4_). Subsequently, the themes changed to “conspiracy theories” and “symptoms and detection” in the stalemate period (T_5_-T_8_), and then progressively concentrated on the themes “prevention action” in the control period (T_9_-T_15_). Finally, in the recover period (T_16_-T_19_), the theme changed to “vaccines and medicines.” This phenomenon is in line with the characteristic that public opinion online would result in a change in themes in a given period [45,46].

Fifth, our study found that the COVID-19 infodemic showed the characteristic of repeated fluctuations. It indicated that the governance of the COVID-19 infodemic on social media is a “protracted-war.” Prior study has also pointed out that the effect of refuting misinformation usually lasts for less than a week [47,48]. Moreover, we found that the repetition rate of the COVID-19 infodemic themes also differed according to the time segments. The theme “conspiracy theories” was significantly more thrive than the themes “symptoms and detection” and “vaccines and medicines.” One possible explanation is that the theme “conspiracy theories” comprised more uncertain knowledge than the themes “symptoms and detection” and “vaccines and medicines.” Therefore, users are more inclined to repeat posts of the theme “conspiracy theories.”

With regard to the practical implications to curb the COVID-19 infodemic on Chinese social media, our findings suggest that the government and health agencies should manage the infodemic in a stage-wise manner and take more efforts to disseminate accurate and professional information via social media to ameliorate the spread of falsehoods. For instance, expert-approved or peer-reviewed videos are expected to provide credible health information. Furthermore, government and health agencies must pay close attention to the spread of the infodemic on video-sharing platforms. Third, they should coordinate with social media companies to establish long-term systems for the prevention and control of the infodemic. For example, social media platforms can curb the repeated dissemination of COVID-19 misinformation by setting alert labels for repeated misinformation and regularly pushing corrective information to users. Additionally, social media may offer novel opportunities for the government and health agencies to assess and predict the trend of epidemic outbreaks.

### Limitations

There are some limitations to this study. First, we targeted posts on Chinese social media; thus, our conclusions may not be applied to social media platforms in other countries, such as Twitter. Second, we collected and analyzed only a relevant subset of all posts about the COVID-19 infodemic, which inevitably introduces some selection bias. Third, as the COVID-19 infodemic continues to disseminate, we should extend the time and expand the data volume to provide the government and health agencies with a more comprehensive prevention and control response. Additionally, our analyses of the repetition of infodemic are still inadequate, and we will further explore this interesting phenomenon in a future study.

### Conclusions

Our study found that the COVID-19 infodemic on Chinese social media was characterized by gradual progress, videoization, and repeated fluctuations. Our findings suggest that the COVID-19 infodemic paralleled the propagation of the COVID-19 epidemic. These findings can help the government and health agencies collaborate with major social media companies to develop targeted measures to prevent and control the COVID-19 infodemic on Chinese social media. Moreover, social media offers a novel opportunity for the government and health agencies to surveil epidemic outbreaks.

## Appendix

Multimedia Appendix 1 Different types of Chinese social media and major social media platforms.

## Abbreviations

LDA: latent Dirichlet allocation
WHO: World Health Organization
