# Supplementary material for: Characterizing the COVID-19 Infodemic on Chinese Social Media: Exploratory Study
Source: JMIR Public Health Surveill. 2021 Feb 5;7(2):e26090. doi: 10.2196/26090 (PMC7869922; doi:10.2196/26090)
Supplement: Multimedia Appendix 1 [file publichealth_v7i2e26090_app1.pdf]

Multimedia Appendix 1: Different types of Chinese social media and major social media platforms.

Chinese social media types	Major social media sites	URL	Brief introduction
**Chat platforms**	WeChat	https://www.wechat.com/	WeChat (Weixin in Chinese), first released in 2011 and became the world's largest standalone mobile application in 2018, with over 1 billion monthly active users. It provides multiple mobile services, including text messaging, hold-to-talk voice messaging, broadcast (one-to-many) messaging, sharing of photographs and videos.
	Weibo	https://www.weibo.com/	Weibo is one of the most popular social media platforms in China and is the equivalent of Twitter in China. It provides Chinese-based mini-blogging services, including social chat sites and platform sharing. The social media platform has more than 500 million users and millions of posts per day, and is adding 20 million new users per month.
	QQ	https://im.qq.com/	QQ is an instant messaging software service and web portal developed by the Chinese tech giant Tencent. It offers services that provide online social games, music, shopping, microblogging, movies, and group and voice chat software. It is the world's 5th most visited website, according to Alexa.
**Video-sharing platforms**	Tiktok	https://www.douyin.com/	TikTok, known in China as Douyin, is a Chinese video-sharing social networking service owned by ByteDance, with over 5 billion monthly active users. The social media platform is used to make a variety of short-form videos, from genres like dance, comedy, and education, that have a duration from 3 to 60 seconds.
	Kuaishou	https://www.kuaishou.com/	Kuaishou is a well-known video-sharing platform in China, with a particularly strong user base among users outside of China's Tier 1 cities.
	Pear Video	https://www.pearvideo.com/	Pear Video is a leading video-sharing platform in China, created by a professional team with a deep media background and a global network of filmmakers, dedicated to providing short video products suitable for mobile viewing and sharing for the younger generation.
**News-sharing platforms**	Toutiao	https://www.toutiao.com/	Toutiao is one of China's largest news-sharing platforms of content creation, aggregation and distribution underpinned by machine learning techniques, with 120 million daily active users as of June 2019.
	Sina news	https://news.sina.com.cn/	Sina news is a Chinese news and information content platform. It provides customized news, entertainment and professional media content for mobile users.
	Tencent news	https://news.qq.com/	Tencent News is a Chinese news-sharing platform that provides users with latest information, video and live broadcast services.
**Health care platforms**	DXY.cn	https://portal.dxy.cn/	DXY.cn is a Chinese online community for physicians, health care professionals, pharmacies and facilities, with more than 3.2 million members.
	Haodf.com	https://www.haodf.com/	Haodf.com is one of the largest Chinese OHCs in which over 300,000 doctors from 3270 regular hospitals in China assist patients by answering questions, treating illnesses and offering emotional support.
	Chunyu Yisheng	https://www.chunyuyisheng.com/	Chunyu Yisheng is an online health community in China. The main purpose of this online community is to connect patients with physicians through a mobile device. It has more than 30 million users who have used it to reach over 40,000 physicians.
**Q&A platforms**	Zhihu	https://www.zhihu.com/	Zhihu is the largest Q&A community in China. It has brought together nearly 70 million users from the Internet and covers diverse fields such as technology, business, psychology, and culture, resulting in 15 million questions asked, 55 million answers given, and 250,000 topics discussed
	Douban	https://www.douban.com/	Douban is one of the largest interest-oriented communities for book, music, and movie reviews. It could be seen as one of the most influential web 2.0 websites in China.
	Jianshu	https://www.jianshu.com/	Jianshu is one of largest knowledge exchange communities in China that allows users to submit questions and answer questions from other users.
